# Synergistic combination of cannabidiol and celecoxib or 2,5-dimethylcelecoxib exerts oxidative stress-mediated cytotoxicity and mitigates glioblastoma invasiveness

**DOI:** 10.3389/abp.2025.15062

**Published:** 2025-09-29

**Authors:** Anna Rybarczyk, Aleksandra Majchrzak-Celińska, Violetta Krajka-Kuźniak

**Affiliations:** ^1^ Chair and Department of Pharmaceutical Biochemistry, Faculty of Pharmacy, Poznan University of Medical Sciences, Poznań, Poland; ^2^ Doctoral School, Poznan University of Medical Sciences, Poznań, Poland

**Keywords:** glioblastoma, cannabidiol (CBD), celecoxib, 2,5-dimethylcelecoxib (2,5-DMC), synergism

## Abstract

Glioblastoma remains one of the most aggressive and treatment-resistant malignancies. Current treatment options, such as radio- and chemotherapy, induce oxidative stress-mediated DNA damage leading to cancer cell death, but are also neurotoxic and not efficient in long term. Our study investigated the effects of cannabidiol, celecoxib and 2,5-dimethylcelecoxib, individually and in combinations, on U-138 MG glioblastoma cell survival, oxidative stress, canonical and non-canonical Nrf2 pathway activation, cell migration and apoptosis. Using the MTT and flow cytometry assay we found that the analyzed compounds and their combinations induce dose-dependent, synergistic, and oxidative stress-related cytotoxicity, with minimal impact (at the concentrations exhibiting anti-cancer effects) on non-cancerous human astrocyte (HA) cell line. The Nrf2 ELISA assay was used for the analysis of the nuclear binding of the nuclear factor-2 erythroid related factor-2 (Nrf2), which followed by the RT-qPCR and Western blot analysis, confirmed the antioxidant response of cells to the applied treatments. Diminished migratory potential, and increase of the autophagy-related p62, LC3 and apoptosis-related caspase-3 protein levels were also observed in response to the treatment with the analyzed compounds. Overall, our study provides evidence that cannabidiol combined with celecoxib or 2,5-dimethylcelecoxib may represent a promising strategy for glioblastoma treatment.

## Introduction

Glioblastoma is the most common and aggressive form of primary brain tumor in adults, characterized by rapid progression, heterogeneity, and resistance to conventional therapies. Despite advances in treatment modalities, including maximal surgical resection, radiation therapy, and alkylating chemotherapy with temozolomide, the prognosis for glioblastoma patients remains dismal, with a median survival of only 12–15 months and a 5-year survival rate of less than 5% (Jaoude et al., 2019). Thus, novel treatment options are needed. One of the possible options for improving therapeutic outcomes in glioblastoma is to create synergistic pro-oxidant therapy, which could be effective in such concentrations that are not harmful to normal cells. Such an approach allows selective targeting of glioblastoma cells without destructive effects on normal astrocytes and neurons.

Oxidative and nitrosative stress, as well as inflammation, contribute to the mechanisms of gliomagenesis, tumor progression and drug resistance (Ostrowski and Pucko, 2022). The rapid growth of glioblastoma cells creates prolonged hypoxia which results in oxidative stress, hindering oxidative phosphorylation and increasing reactive oxygen species (ROS) production in the mitochondria. This leads to the reprogramming and development of glioblastoma stem cells (GSC). These cells in particular show signs of high oxidative stress, partly because of their high metabolic demand and hypoxic conditions (Schroeder et al., 2020). In response to variations in oxygen and ROS levels, nuclear respiratory factors and hypoxia-inducible factors are activated, all of which start tumor metabolic reprogramming (Krawczynski et al., 2020). It is also the Achilles’ heel of glioblastoma cells, because the pro-oxidative therapies, such as radio- and chemotherapy, further overload the mitochondria and intracellular environment with too many ROS, ultimately leading to apoptosis. In fact, the majority of anticancer treatments and targeted therapies, function by either directly or indirectly producing ROS (Farhan, 2024). Therefore, the pro-oxidant effects of new drug regimens are of high value. On the other hand, the safety of such new therapies to normal astrocytes and neurons is crucial, as those cells are prone to oxidative stress-induced damage, which can lead to neurocognitive side effects.

In this regard, plant-derived agents present an interesting option, as they possess antioxidant properties, but can also exhibit pro-oxidant behavior, depending on the context (Farhan, 2024). In contrast to synthetic chemotherapeutics, such as temozolomide, plant-derived compounds possess unique anticancer properties and very often high selectivity for tumor cells, without affecting normal cells (Farhan, 2024; Majchrzak-Celińska and Studzińska-Sroka, 2024). In this context, cannabidiol (CBD) have been found to exert interesting anticancer effects, which are accompanied by its neuroprotective and anti-inflammatory properties (Rybarczyk et al., 2023a). CBD is a multi-mechanism agent that modulates many signaling pathways in cancer cells and is considered a non-opioid analgesic. Recently, CBD is being evaluated in clinical trials as an anti-glioblastoma agent (Rybarczyk et al., 2023b; Feng et al., 2024). On-going reports provide evidence of very promising results which rises hopes that the implementation of CBD as a new anti-glioblastoma agent can be expected in the nearest future.

Moreover, it is important to consider neuroinflammation as a key factor contributing to gliomagenesis, when thinking of new anti-glioblastoma therapies. Neuroinflammation is promoted by hypoxic tumor microenvironment, increased oxidative stress, and an immune suppressive milieu (Alghamri et al., 2021). Glioblastoma cells express and secrete immune suppressive chemokines and cytokines, including interleukin (IL)-6, IL-10, transforming growth factor (TGF)-β, and galectin-1, which act on infiltrating immune cells to hijack them by inducing a protumor cellular phenotype (Roesler et al., 2021). Tumor cells also overexpress cyclooxygenase-2 (COX-2) and its major product within the brain, prostaglandin E2 (PGE2) (Qiu et al., 2017; Dean and Hooks, 2023). The latter acts on its E-prostanoid (EP) receptors to regulate cell proliferation, migration, apoptosis, and angiogenesis, contributing to tumor progression (Qiu et al., 2017). In our previous study we showed that COXIBs, in particular celecoxib and 2,5-dimethylcelecoxib (2,5-DMC), counteract the hyperactivated Wnt pathway and COX2/PGE2/EP4 pathway, providing therapeutic benefit both when used alone or in a combination with temozolomide (Majchrzak-Celińska et al., 2021). Moreover, our most recent study showed that combining tinostamustine with celecoxib more effectively induces apoptosis and halts cellular migration, as compared to tinostamustine alone (Pawlak and Majchrzak-Celińska, 2025). Together with mounting evidence for the benefits of COXIBs in glioblastoma treatment, there is also a growing need for further exploration of possible other drug combinations involving COXIBs, which might accelerate glioblastoma research.

Thus, this study aimed to examine whether CBD + celecoxib and CBD + 2,5-DMC have a higher anti-glioblastoma efficacy as compared to individual compounds. In this regard we evaluated their cytotoxicity against U-138 MG glioblastoma cell line and human astrocyte HA cell line. We also explored the involvement of ROS production and the antioxidant Nrf2 response with canonical and non-canonical pathways. Nrf2 nuclear translocation and binding, followed by the analysis of Nrf2-pathway downstream proteins were applied to verify the cellular response to applied treatments. Finally, the impact of the analyzed compounds and their combinations on cellular motility, autophagy and apoptosis was evaluated.

## Materials and methods

### Cell culture

The cell lines used were glioblastoma U-138 MG purchased from American Type Culture Collection (ATCC, Gaithersburg, MD, United States) and human astrocytes (HA) purchased from ScienCell (Carlsbad, CA, United States). U-138 MG cells were maintained in Eagle’s Minimum Essential Medium EMEM (Gibco, ThermoFisher Scientific, Waltham, MA, United States) supplemented with 10% fetal bovine serum (Merck, Darmstadt, Germany) and 1% penicillin/streptomycin antibiotics (Merck, Darmstadt, Germany). Astrocytes were cultivated as monolayers in Corning^®^ BioCoat™ Poly-D-Lysine Cell Culture Flask in Astrocyte Medium (ScienCell, Carlsbad, CA, United States) supplemented with 2% FBS, 1% Astrocyte Growth Supplement and penicillin/streptomycin 1% solution. Both cell lines were kept at 37 °C in a humidified atmosphere with a 5% CO_2_ environment.

### Compounds

Cannabidiol (CBD) was purchased from Medcolcanna Organics Inc. (Distrito Especial, Colombia). Celecoxib and 2,5-dimethylcelecoxib (2,5-DMC) were obtained from Merck (Darmstadt, Germany). Doxorubicin (DOXO) was applied at 500 nM as a positive control in the experiments analyzing ROS generation and apoptosis. Dimethyl sulfoxide (DMSO) served as a negative control and a solver of all tested compounds. The final DMSO concentration in all experiments was 0.1% (v/v) and showed no effect on U-138 MG or HA viability in preliminary tests.

### Viability assay

To perform the cytotoxicity test, we used colorimetric MTT assay which is based on mitochondrial succinate dehydrogenase activity to convert tetrazolium salts into a colored formazan in metabolically active cells. U-138 MG and HA cells were seeded into sterile 96-well plates, pre-coated with poly-D-lysine for HA. The cell density was adjusted to 1x10^4^ cells per well. Following a 24-h incubation, cells were exposed to CBD, celecoxib, 2,5-DMC, and their combinations at concentrations between 1 and 100 μM and maintained over 24 h. The media were discarded and cells were washed with 200 µL PBS before incubation with 200 μL of MTT solution (5 mg/mL) at 37 °C for 4 h. By shaking the plates for 15 min, the formazan was dissolved in 200 µL of acidic isopropanol. The absorbance at wavelengths of 570 and 690 nm was measured using the Biotech TECAN Infinite M200 reader (Grödig, Austria). The cell viability was calculated as a percentage of the absorbance obtained from the treated cells in comparison to the absorbance from DMSO-treated control. All experiments were repeated three times.

### Synergy assesment

To evaluate synergy between CBD and celecoxib, and CBD and 2,5-DMC, we processed the results of cytotoxicity test (MTT assay) using SynergyFinder Plus open-access web application (accessed on 28 February 2025)^1^ (Zheng et al., 2022). The raw dose-response data were uploaded and calculated using the Zero Interaction Potency (ZIP) Loewe, Bliss and HSA models. The synergy scores were visualized via 2D contour plots to assess whether drug combinations exhibited synergistic, additive, or antagonistic effects. Drug combinations with synergy scores over 10 demonstrate synergy, a score between −10 and 10 implies an additive relationship, whereas antagonistic interactions appear when the score drops below −10.

### Oxidative stress assay

To evaluate intracellular superoxide radicals level, the manufacturer’s guidelines for the Muse^®^ Oxidative Stress Kit (Merck, Darmstadt, Germany) were followed. The method is based on dihydroethidium (DHE), the reagent which penetrates the cell membrane and interacts with superoxide anions. Upon oxidation, ethidium bromide, a DNA-binding agent, is produced and emits fluorescence. Briefly, U-138 MG glioma cells (3 × 10^5^ per well) were cultured in 6-well plates for 24 h. Following 24 h of treatment with the compounds, cells were washed with PBS and resuspended in 1X Assay Buffer to reach a final concentration of 1 × 10^6^ to 1 × 10^7^ cells/mL. The cell suspension (10 µL) was mixed with 190 µL of working solution containing the diluted Muse^®^ Oxidative Stress Reagent. After gentle mixing, cells were incubated at 37 °C for 30 min before being analyzed using the Muse^®^ Cell Analyzer (Merck, Darmstadt, Germany). Data were evaluated using Muse^®^ Analysis Software ver. 1.4 (Merck, Darmstadt, Germany).

### Apoptosis analysis

The U-138 MG cells were seeded into 12-well plates at 5 × 10^4^ per well and incubated overnight. Then, the cells were treated with tested compounds and combinations for 24 h. Using the Annexin V and Dead Cell kit (Merck, Darmstadt, Germany), apoptosis assay was carried out according to the manufacturer’s protocol. After 20 min of staining with reagents containing annexin V and 7-amino-actinomycin D (7-AAD), cells were analyzed on the Muse^®^ Cell Analyzer (Merck, Darmstadt, Germany). The obtained data were processed using the Muse^®^ 1.4 Analytical Software (Merck, Darmstadt, Germany).

### Cytosol/nuclear fractionation

To obtain cytosolic and nuclear cellular protein extracts, U-138 MG cells were seeded into 6-well plates at a density of 3 × 10^5^ per well. Following adherence of the cells within 24 h of incubation, CBD, celecoxib, 2,5-DMC in 5 and 10 µM concentrations were added as well as their combinations at the corresponding concentrations. The cytosolic and nuclear fractions were isolated according to the Nuclear/Cytosol Fractionation Kit protocol (Abcam, Cambridge, England).

### Western blot analysis

Protein quantification was performed using the Lowry method. Equal amounts of protein were separated on 7.5%, 10% or 12% SDS gels and transferred to a Immobilon P membrane (Sigma-Aldrich, St. Louis, MO, United States). After blocking with 10% skimmed milk for 2 h, the Immobilon P membranes were incubated overnight with the following primary antibodies against: Nrf2 (sc-365949, Santa Cruz, CA, United States), SOD-1 (sc-8637, Santa Cruz, CA, United States), CAT (sc-34285, Santa Cruz, CA, United States), p62/SQSTM1 (sc-28359, Santa Cruz, CA, United States), caspase-3 (sc-271028, Santa Cruz, CA, United States), mTOR (7C10, Cell Signalling, Danvers, MA, United States), LC3 (sc-398822, Santa Cruz, CA, United States), β-actin (sc-47778, Santa Cruz, CA, United States) and lamin A (sc-20680, Santa Cruz, CA, United States). β-actin and lamin A served as loading controls. Following triple washes, the membranes were exposed to appropriate HRP-conjugated secondary antibodies. Visualization of bands was achieved using the chemiluminescent HRP substrates from Clarity ECL Kits (Bio-Rad Laboratories, Hercules, CA, USA). The results were presented in relative absorbance units (RQ) per mg of protein and expressed as a fold of the control. The total densitometry of all blot-transferred protein bands per lane was estimated by Image Lab 6.1.0 software (BioRad Laboratories, Hercules, CA, United States).

### Real-time quantitative PCR (RT-qPCR)

Glioblastoma U-138 MG cells seeded into 6-well plates (2 × 10^5^ per well) were treated with compounds for 24 h. The GeneMatrix Universal DNA/RNA/Protein Purification Kit (EURx, Gdańsk, Poland) was used to isolate total RNA. Quantitative analysis was performed by Nanodrop ND-1000 Spectrophotometer (ThermoFisher Scientific, Waltham, MA, United States). Single-stranded cDNA was synthesized with the Revert-Aid First Strand cDNA Synthesis kit (Fermentas, Burlington, Canada) while the SYBR^®^ Green Master Mix kit was utilized for RT-qPCR. The analysis was performed using the LightCycler96 version 1.1.0.1320 (Roche, Basel, Switzerland). The protocol initiates pre-incubation as initial denaturation at 95 °C for 10 min, followed by 40 cycles of denaturation (95 °C for 15 s), annealing (56 °C for 30 s) and extension (72 °C for 30 s). Primers were acquired from oligo.pl (Warsaw, Poland), and their specific sequences are listed in Table 1. For normalization, the expression of *TBP* and *PBGD* was utilized.

**TABLE 1:** The sequences of primers used in RT-qPCR.

Gene	Forward primer	Reverse primer
*Nrf2*	5′ATT​GCT​ACT​AAT​CAG​GCT​CAG	5′GTT​TGG​CTT​CTG​GAC​TTG​G
*CAT*	5′TGG​ACA​AGT​ACA​ATG​CTG​AG	5′TTA​CAC​GGA​TGA​ACG​CTA​AG
*SOD1*	5′CGA​CAG​AAG​GAA​AGT​AAT​G	5′TGG​ATA​GAG​GAT​TAA​AGT​GAG​G
*TBP*	5′GGC​ACC​ACT​CCA​CTG​TAT​C	5′GGG​ATT​ATA​TTC​GGC​GTT​TCG
*PBGB*	5′TCA​GAT​AGC​ATA​CAA​GAG​ACC	5′TGG​AAT​GTT​ACG​AGC​AGT​G

### Nrf2 binding assay

The TransAM™ Nrf2 ELISA-based kit (Active Motif, Carlsbad, CA, USA) was applied to determine Nrf2 binding activity in U-138 MG cells. Oligonucleotide-coated 96-well plates, designed with the Nrf2/ARE consensus sequence (5′-GTC​ACA​GTG​ACT​CAG​CAG​AAT​CTG-3′) were incubated with nuclear fraction diluted in Complete Lysis Buffer for 1 h at room temperature. Non-specifically bound proteins were removed through extensive washing steps. Subsequently, a primary antibody specific to active Nrf2 at a 1:1000 dilution was added for 1 h. Following additional washing, a horseradish peroxidase (HRP)-conjugated secondary antibody was applied for 1 h to enhance detection sensitivity. A colorimetric reaction was developed and absorbance was measured at 450 nm and 655 nm as the reference wavelength using the Biotech TECAN Infinite M200 reader (Grödig, Austria). All measurements were performed in triplicates.

### Wound healing assay

To assess the migration capacity of U-138 MG cells after exposure to tested compounds, we seeded cells into 96-well plates at 5 × 10^4^ density for reaching ∼95% confluence after 24 h. Cell monolayers were scratched using a sterile pipette tip. Subsequently, wells were washed with PBS in order to remove cell debris and treated with 5 μM and 10 µM drug concentrations. The scratches were photographed under the Millicell^®^ DCI Digital Cell Imager (Merck, Darmstadt, Germany) at 10 magnifications at the time 0 and at 24 and 48 h intervals. The average area of the wound was measured using ImageJ software version 1.8.0. The experiment was repeated 2 times with 4 wells per analyzed compound/s, per assay. After pooling the data from many wells, the final migration area for each test group was subtracted from the average premigration area of the control samples to determine the overall amount of migration.

### Statistical analysis

The statistical analysis to determine significant differences relative to control treatment was performed using one-way ANOVA, post-hoc Dunnett’s test GraphPad Instat version 3.10 (GraphPad Software, San Diego, CA, United States). Values of p < 0.05 were considered significant.

## Results

### CBD with celecoxib and CBD with 2,5-DMC at 5 and 10 µM concentration exhibit synergistic cytotoxicity towards U-138 MG cells, while being safe to human astrocyte HA cell line

**FIGURE 1:**
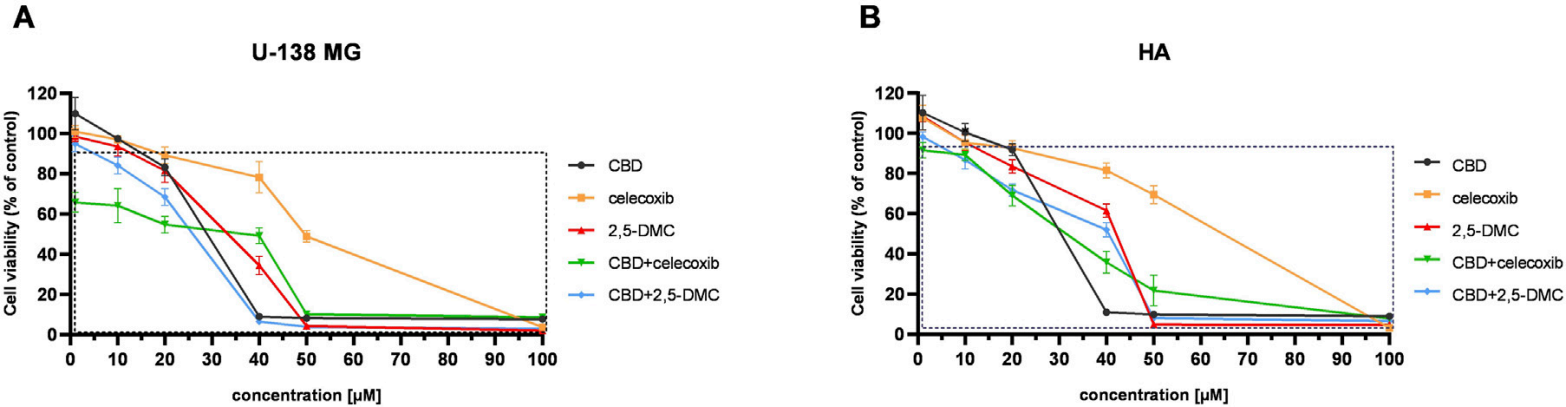
The results of the MTT assay following 24 h exposure of U-138 MG glioblastoma **(A)**, and human astrocyte HA cell line **(B)** to CBD, celecoxib, 2,5-DMC and their combinations. The square indicates statistically significant results as compared to the DMSO-treated control. Data are presented as mean values ± SEM from three independent experiments.

The MTT test performed in a concentration range of 1–100 μM revealed that CBD, celecoxib, 2,5-DMC, and their combinations induce stronger dose-dependent cytotoxicity towards U-138 MG cells, as compared to HA cell line (Figure 1). In this regard, the most cytotoxic drug combination was CBD and celecoxib. This drug combination significantly reduced the number of living cancer cells in all analyzed concentrations, with a dramatic drop of cellular viability at 50 µM concentration. Importantly, the lowest tested concentrations (up to 10 µM), already toxic to glioblastoma cells, were tolerable to HA cells, with only around 10% of them dying in these conditions. Moreover, the second tested drug combination, i.e., CBD and 2,5-DMC, exhibited very similar pattern, with more pronounced changes in cellular response in 40 µM concentration; U-138 MG cells were totally dead in this concentration, while more than 50% of astrocytes was still alive in these conditions. Regarding single compounds treatment, CBD was more toxic than celecoxib and 2,5-DMC, with a similar viability pattern to the CBD and 2,5-DMC combination in U-138 MG cells. In HA astrocyte cells this compound already at 40 µM caused massive cell death. Our results also revealed that 2,5-DMC and celecoxib in a concentration range of 1–20 µM had similar effects on cellular viability in both cell lines, while in higher concentrations (ranging from 20–50 µM), celecoxib showed lower cytotoxicity as compared to 2,5-DMC. In a concentration of 100 µM all the analyzed compounds and combinations were toxic to both cell lines. The IC50 values obtained after the MTT assay are presented in Table 2.

**TABLE 2:** The IC_50_ values for U-138 MG glioblastoma and HA human astrocyte cell lines, following 24 h incubation with the analyzed compounds or their combinations.

Compounds and combinations	IC_50_ [µM]	
	U-138 MG	HA
CBD	23.95 ± 0.90	24.98 ± 0.32
Celecoxib	49.49 ± 0.84	64.57 ± 2.56
2,5-DMC	35.78 ± 4.86	40.77 ± 0.15
CBD + celecoxib	41.81 ± 0.36	30.33 ± 2.64
CBD + 2,5-DMC	24.32 ± 0.67	35.95 ± 4.43

Based on the MTT results, and following the fundamental principle of tolerable toxicity towards normal cells, subsequent experiments were carried out at 5 and 10 µM concentrations over a 24-h exposure period. In the case of drug combinations, 5 µM total concentration equaled 2.5 µM of one compound and 2.5 µM of the other compound, while 10 µM concentration equaled 5 µM of one compound and 5 µM of the other compound.

**FIGURE 2:**
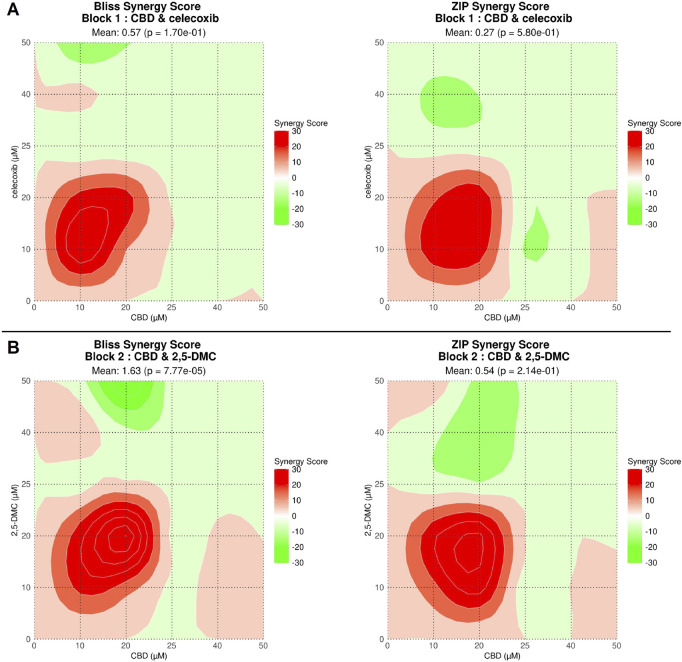
Drug combination dose-response data obtained using SynergyFinder Plus tool for CBD + celecoxib **(A)** and CBD + 2,5-DMC combinations **(B)**. Drug combinations with synergy scores over 10 demonstrate synergy (indicated with intensive red color), a score between −10 and 10 implies an additive relationship (indicated with fade red and green colors or white), whereas antagonistic interactions appear when the score drops below −10 (indicated with intensive green color).The synergy scores reveal CBD and celecoxib and CBD and 2,5-DMC synergistic cytotoxicity in a concentration range of 1–25 μM, with the most pronounced synergism for a concentration range of 10–20 µM for both tested combinations.

In order to obtain drug combination dose-response data we performed the computational analysis using SynergyFinder Plus tool (Figure 2). The results revealed that both drug combinations, i.e., CBD and celecoxib as well as CBD and 2,5-DMC exhibit similar synergism pattern. Both drug combinations have a synergistic mode of action in the concentrations up to ∼25 µM each, with the most pronounced synergism in a concentration range of 10–20 µM. Combinations in higher concentrations of CBD and celecoxib/2,5-DMC exhibited mostly additive effects. As reflected in Figure 2, the mixes in some specific concentrations had antagonistic effects.

### CBD, celecoxib and 2,5-DMC induce oxidative stress in U-138 MG glioblastoma cells with very similar ROS profiles as a response to single compound vs. combinatory treatment

**FIGURE 3:**
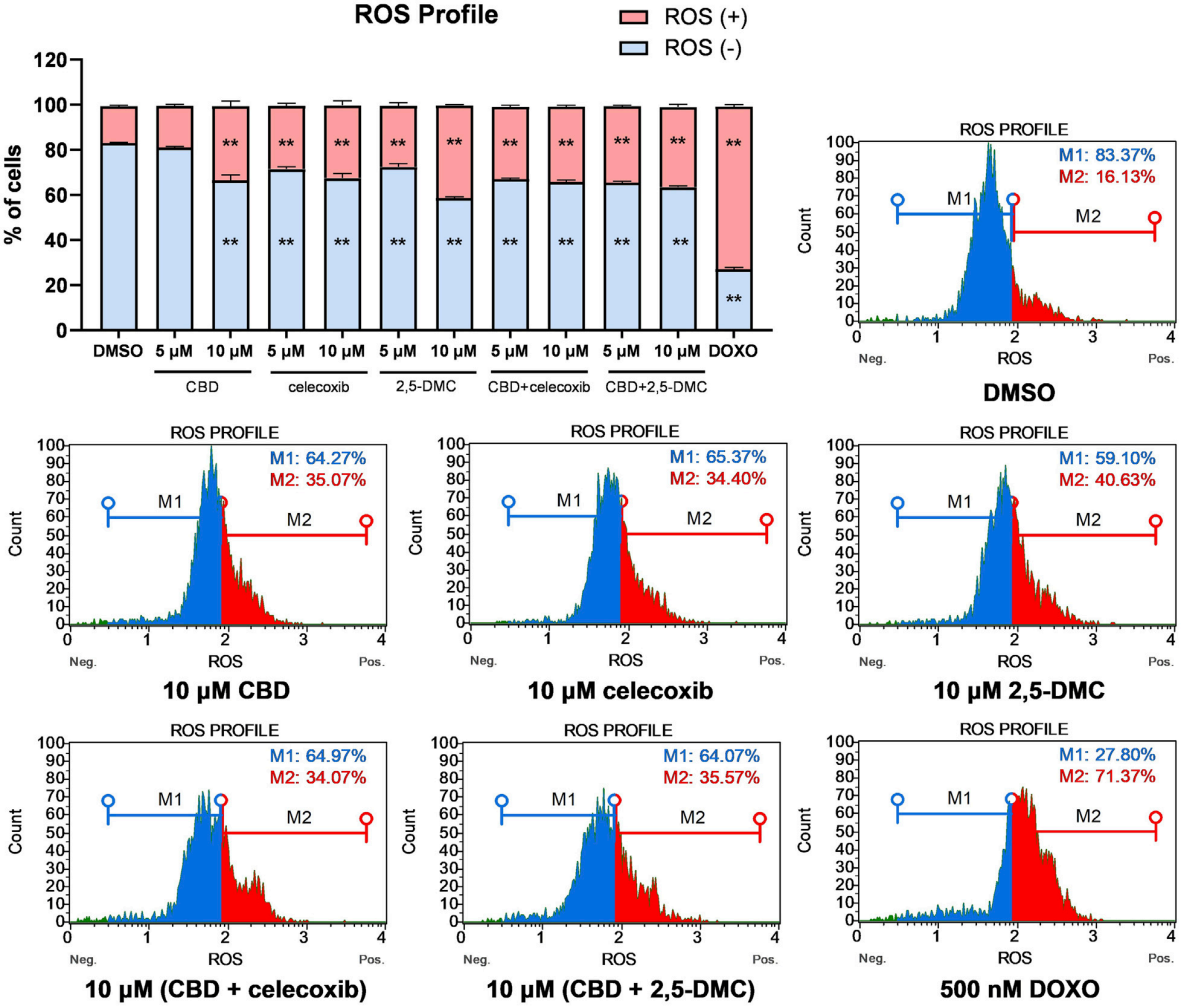
ROS profile obtained in U-138 MG cells after the 24 h treatment with the analyzed compounds and their combinations. DMSO and doxorubicin (500 nM) served as negative, and positive control of the assay, respectively. ROS (+) and ROS (−) indicate cells with detected and undetected superoxide radicals, respectively. Values are expressed as mean ± SEM from two independent experiments run in duplicate. Double asterisk (**) indicates the values significantly different from the DMSO-treated control with p < 0.01. Representative histograms are also presented.

Next, we wanted to verify whether oxidative stress mediates the cellular toxicity of the analyzed compounds and their combinations in U-138 MG glioblastoma cells. Our flow cytometry results showed that all the analyzed compounds and their combinations at both 5 μM and 10 µM concentrations induce significant oxidative stress in glioblastoma cells, with around twice as much ROS (+) cells in the treated cells as compared to DMSO-treated negative control cells (Figure 3). Overall, we did not observe more pronounced oxidative stress when the drug combinations were used, as compared to single compounds.

### CBD, celecoxib and 2,5-DMC exert pro-apoptotic effect in glioblastoma cells

**FIGURE 4:**
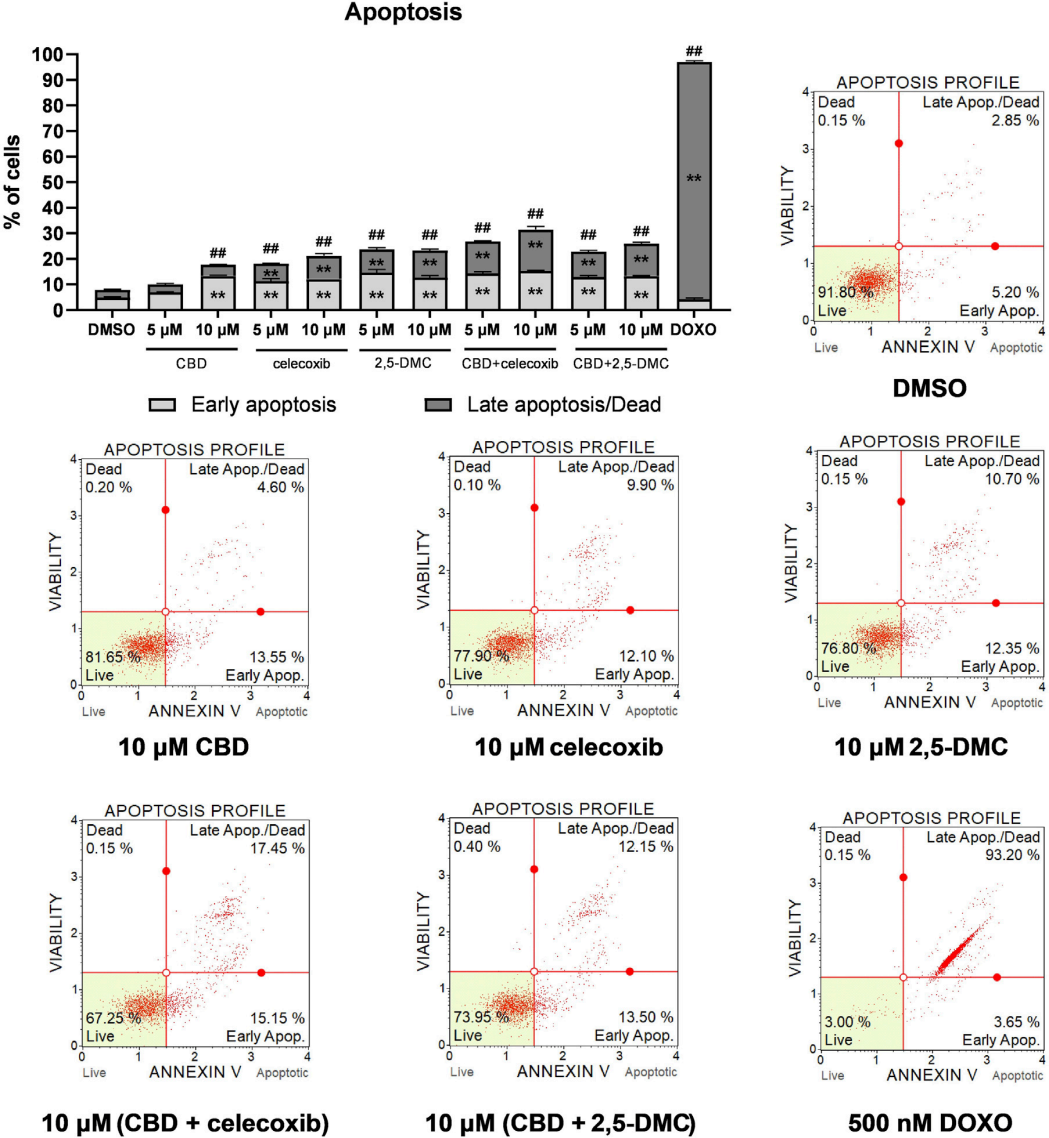
Apoptosis of U-138 MG cells treated with CBD, celecoxib and 2,5-DMC, and their combinations for 24 h and analyzed using Muse ™ Annexin V and Dead Cell Kit (Merck, Darmstadt, Germany). DMSO and doxorubicin (500 nM) served as negative, and positive control of the assay, respectively. Values are expressed as mean ± SEM from two independent experiments run in duplicate. Double asterisk (**) indicates the values significantly different from the DMSO-treated control with p < 0.01. A hashtag (#) above the bar indicates statistically significant differences as compared to the DMSO-treated control for total apoptotic cells. Representative histograms are also presented.

Next, we wanted to verify if oxidative stress increase induced by the treatment with CBD, celecoxib and 2,5-DMC triggers apoptosis in U-138 MG cells. The results of flow cytometry assay indicate, that in all cases the total number of apoptotic cells is higher as compared to the DMSO-treated negative control cells (Figure 4). Generally, the highest pro-apoptotic effect was observed after the treatment with a mixture of CBD and celecoxib in 10 µM concentration.

**FIGURE 5:**
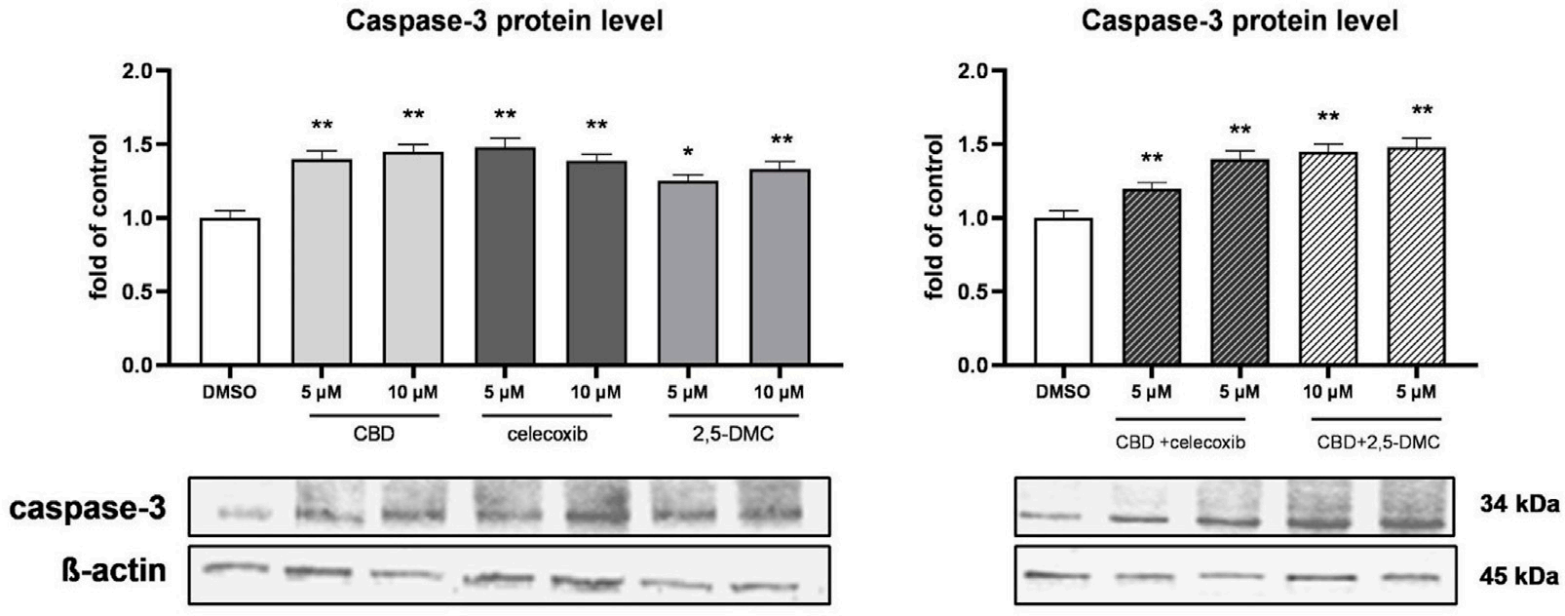
Western blot analysis of caspase-3 protein level in the cytosol of U-138 MG cells treated with CBD, celecoxib and 2,5-DMC and their combinations. Values are expressed as mean ± SEM from two independent experiments run in duplicate. Single asterisk (*) and double asterisk (**) indicate the values significantly different from the DMSO-treated control (level equal to 1) with p < 0.05, and p < 0.01, respectively.

Additionally, in order to analyze the pro-apoptotic effects of the analyzed compounds and their combinations, we analyzed the level of caspase-3 protein in the cytosolic fraction of U-138 MG cells, after the treatment with CBD, celecoxib and 2,5-DMC (Figure 5). Our results show increase in the level of caspase-3 following the 24 h of incubation with all the analyzed compounds and their combinations, confirming their pro-apoptotic properties towards glioblastoma cells.

### Canonical and non-canonical Nrf2 pathways are involved in the cellular response to CBD, celecoxib and 2,5-DMC treatment of U-138 MG cells

**FIGURE 6:**
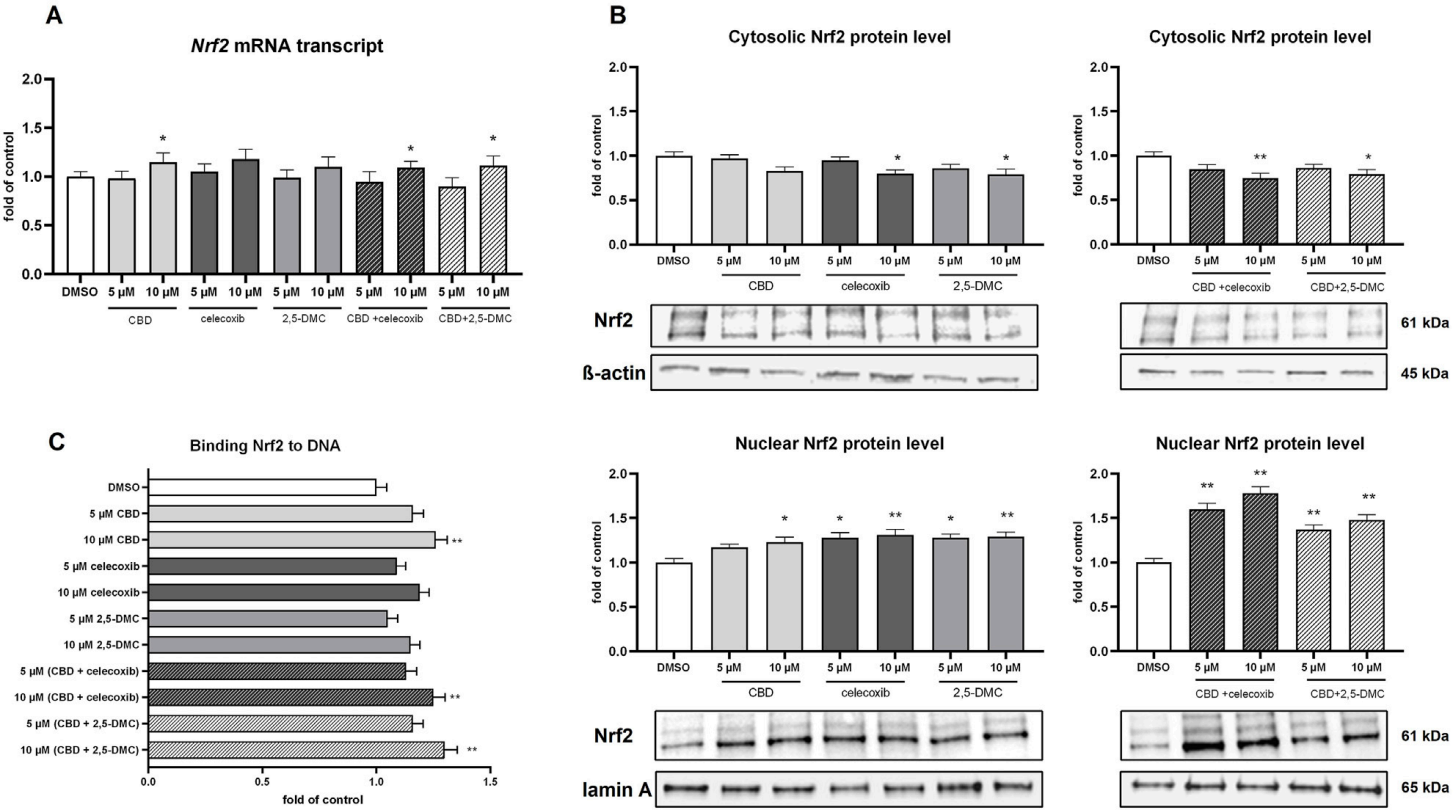
The impact of the analyzed compounds on canonical and non-canonical Nrf2 pathway in U-138 MG cells following 24 h incubation. **(A)** Results of the RT-PCR analyzing the expression of Nrf2 at the mRNA level. **(B)** Western blot results of the Nrf2 protein level in the cytosol and nucleus. **(C)** Results of the ELISA colorimetric assay detecting Nrf2 biding affinity to DNA. Values are expressed as mean ± SEM from two independent experiments run in duplicate. Single asterisk (*) and double asterisk (**) indicate the values significantly different from the DMSO-treated control (expression or level equal to 1) with p < 0.05, and p < 0.01, respectively.

Subsequently, using qRT-PCR, Western blot and ELISA assay we analyzed how the U-138 MG cells respond, in terms of the Nrf2 signaling, to the 24 h treatment with the analyzed compounds and their combinations. The results of our study revealed that despite the induction of the oxidative stress in the cells, the expression of Nrf2 at the mRNA level remains relatively stable (Figure 6A). Only in the case of 10 µM CBD and its combinations with celecoxib and 2,5-DMC, the expression of Nrf2 was slightly, but significantly upregulated. Nevertheless, we observed the increased level of Nrf2 protein in the nucleus (as a response to all treatments tested, except 5 µM CBD), which was accompanied by the decreased level of Nrf2 in the cytosol (in the case of 10 µM celecoxib and its equimolar combination with CBD, as well as 10 µM CBD and 2,5-DMC), indicating a nuclear translocation of this transcription factor following the treatment (Figure 6B). The ELISA assay, however, confirmed the increased binding of Nrf2 to the Nrf2/ARE consensus sequence only in the case of 10 µM CBD, 10 µM (CBD + celecoxib) and 10 µM (CBD + 2,5-DMC) treatment (Figure 6C). In all other cases the DNA binding affinity of Nrf2 was similar to that measured in the DMSO-treated control.

**FIGURE 7:**
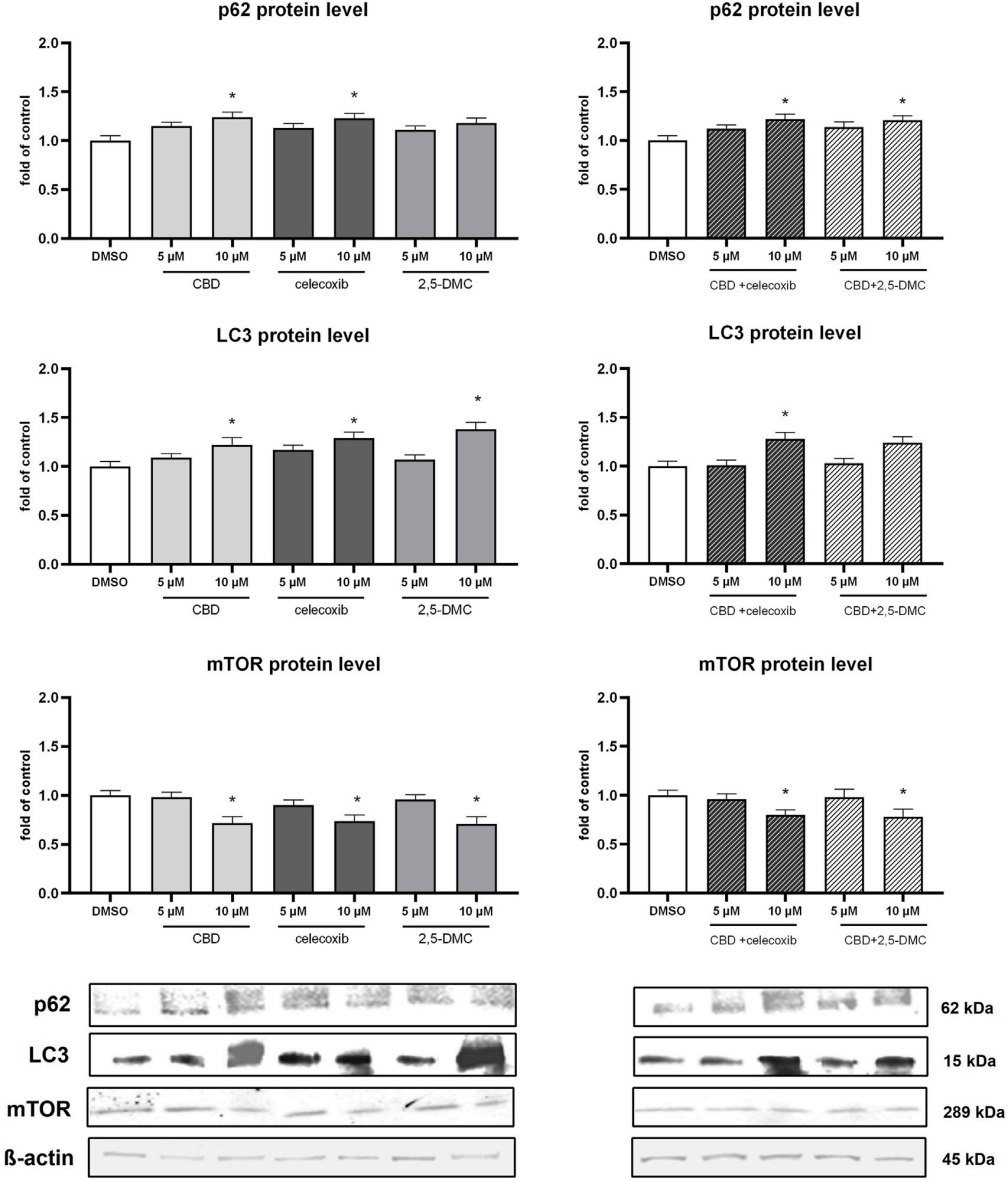
Results of Western blot analysis of p62, LC3 and mTOR protein level in the cytosol of U-138 MG cells following 24 h of treatment with CBD, celecoxib and 2,5-DMC and their combinations. Values are expressed as mean ± SEM from two independent experiments run in duplicate. Single asterisk (*) indicates the values significantly different from the DMSO-treated control (level equal to 1) with p < 0.05.

In order to verify if the analyzed compounds and their combinations have impact on non-canonical Nrf2 pathway, we analyzed p62 protein level in the cytosol of U-138 MG cells (Figure 7). Our results provide evidence, that p62 level increases in response to 10 µM CBD, 10 µM celecoxib, 10 µM (CBD + celecoxib) and 10 µM (CBD + 2,5-DMC) treatment. Furthermore, we assessed LC3 protein as a marker of autophagy (Figure 7). The treatments, particularly with 10 µM CBD, celecoxib, 2,5-DMC and 10 µM (CBD + celecoxib), resulted in accumulation of LC3, suggesting an induction of autophagic processes in U-138 MG cells. In parallel, we examined the mTOR protein, a key negative regulator of autophagy (Figure 7). We observed a reduction in mTOR levels under conditions where LC3 accumulation was most prominent, further supporting the involvement of autophagy in the cellular response to these compounds.

### The level of SOD1, but not CAT, is increased after the treatment with CBD, celecoxib and 2,5-DMC and their combinations

**FIGURE 8:**
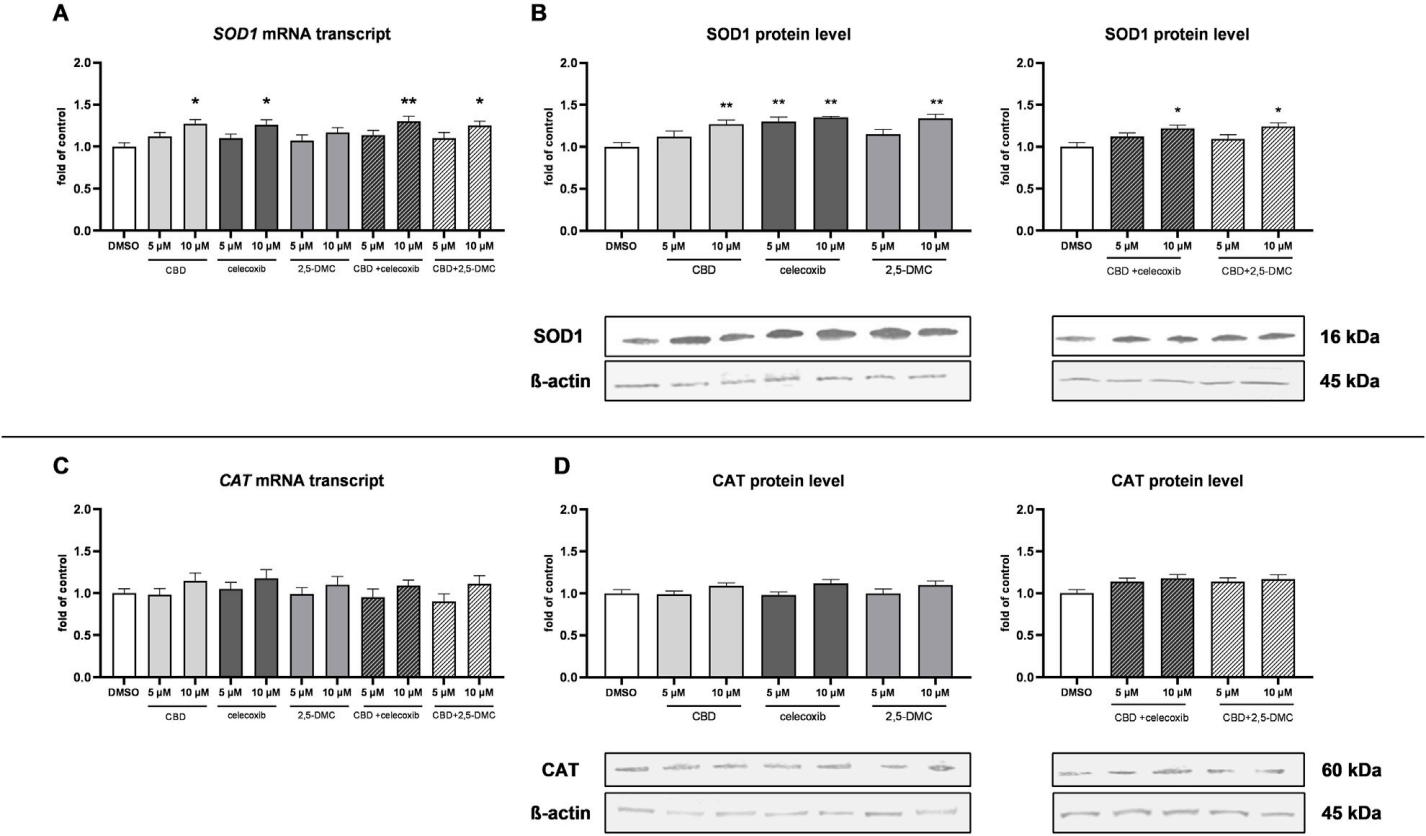
The results of SOD1 and CAT expression analyzed at the mRNA and protein level in the cytosol of U-138 MG cells following 24 h of treatment with CBD, celecoxib and 2,5-DMC and their combinations. **(A)** Results of the RT-PCR analyzing the expression of SOD1 at the mRNA level. **(B)** Western blot results of the SOD1 protein level in the cytosol isolated from cells following the single compounds treatment and combinatory treatment. **(C)** Results of the RT-PCR analyzing the expression of CAT at the mRNA level. **(D)** Western blot results of the CAT protein level in the cytosol isolated from cells following the single compounds treatment and combinatory treatment. Representative immunoblots are shown. Values are expressed as mean ± SEM from two independent experiments run in duplicate. Single asterisk (*) and double asterisk (**) indicate the values significantly different from the DMSO-treated control (expression or level equal to 1) with p < 0.05 and p < 0.01, respectively.

Next, we wanted to verify if the upregulation of Nrf2-dependent antioxidant enzymes partially neutralizes the oxidative stress-mediated cytotoxic effect generated by CBD, celecoxib, and 2,5-DMC. In this regard, we analyzed the mRNA and protein levels of superoxide dismutase 1 (SOD1) and catalase (CAT). Our results revealed, that indeed, the level of SOD1 is increased as a result of the treatment with 10 µM CBD, 5 µM and 10 µM celecoxib and 10 µM 2,5-DMC, and both tested drug combinations in the 10 µM concentration (Figure 8). On the other hand, the expression of CAT, both at the mRNA and protein level, was not altered as a result of the treatments (Figure 8).

### The combinatory treatment of CBD and celecoxib, and CBD and 2,5-DMC strongly halts glioblastoma cell migration

**FIGURE 9:**
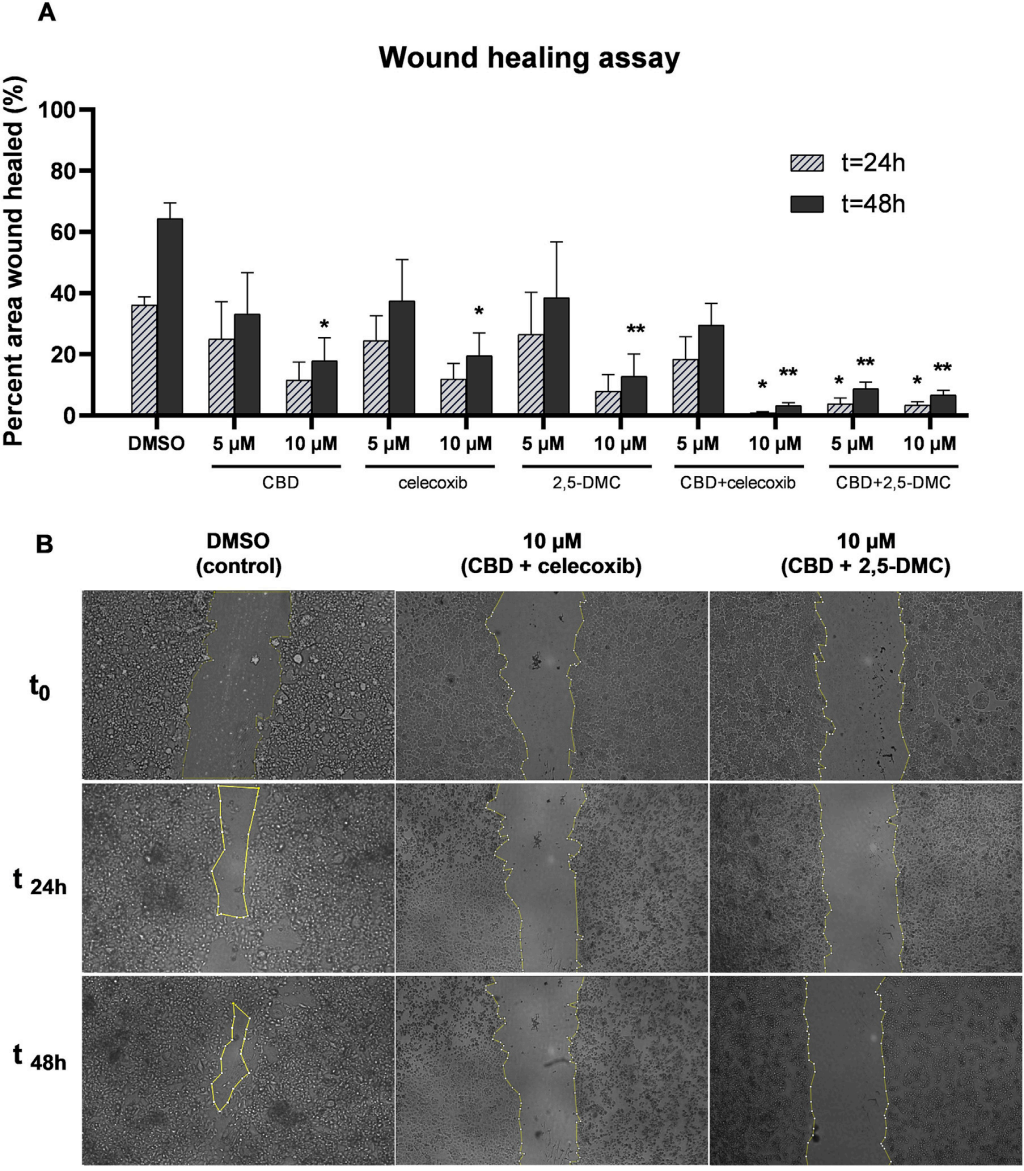
The inhibition of migratory potential of U-138 MG glioblastoma cells as a result of treatment with CBD, celecoxib and 2,5-DMC and their combinations, demonstrated using wound healing assay performed after 24 and 48 h. **(A)** Pooled data from two independent experiments with four measurements per assay. The results from each time point were statistically evaluated in respect to the data obtained from the DMSO-treated control. Single asterisk (*) and double asterisk (**) indicate the values significantly different from the DMSO-treated control with p < 0.05 and p < 0.01, respectively. **(B)** The representative images of the wound healing assay for the control cells and cells treated with drug combinations, exhibiting the most potent anti-migratory potential.

Glioblastoma is characterized by diffuse growth and high invasiveness, therefore we wanted to verify whether the tested compounds influence the migratory potential of cells. In order to do that we performed the wound healing assay and analyzed the data after 24 and 48 h. Our results revealed that after 48 h, all tested compounds and their combinations, when used at a concentration of 10 µM significantly halt the migration of U-138 MG cells (Figure 9). Also, at the lower tested concentration, i.e. 5 μM, the combination of CBD + 2,5-DMC, significantly limited the invasiveness of cells in the first 24 h following the treatment.

## Discussion

This study proposes a new, dual therapy, consisting of CBD with either celecoxib or 2,5-DMC, as an effective anti-glioblastoma treatment inducing oxidative stress-related cytotoxicity, apoptosis and diminishing cellular motility.

Combination therapy, a treatment modality that combines two or more therapeutic agents, is nowadays a cornerstone of cancer therapy (Mokhtari et al., 2017). It is a way to increase the anticancer effect due to synergistic or additive effects exerted by compounds. Recently, the synergistic inhibitory effect of celecoxib and 2,5-DMC plus paclitaxel or cisplatin on human cervix HeLa and SiHa cells was reported (Robledo-Cadena et al., 2024). Previously, we also found that the combination of CBD with ibuprofen or diclofenac brings therapeutic benefit concerning vulvar squamous cell carcinoma (Krajka-Kuźniak et al., 2022). Additionally, regarding the glioblastoma research, our group recently developed poly (lactic-co-glycolic acid)-based nanoparticles loaded with etoricoxib and CBD, which exhibited synergistic effect against two glioblastoma cell lines (Kuźmińska et al., 2023). In this study, we provide evidence that CBD and celecoxib, both at low molar concentrations (up to ∼25 µM), exert synergistic cytotoxic effect against glioblastoma cells. It is important to note, that both compounds cross the blood-brain barrier, so they can potentially exert their synergistic action within the tumor mass (Novakova et al., 2014; Zhu et al., 2018; Calapai et al., 2020). Similar syngery profile was found for the second tested drug combinations, i.e., CBD + 2,5-DMC, in the low molar concentrations (up to ∼25 µM).

In our study we found that the analyzed compounds exert their cytotoxic actions through the induction of oxidative stress. Although initially appearing counterintuitive, due to the association of oxidative stress with malignant transformation, it remains clear, that the induction of oxidative stress in glioblastoma cells brings therapeutic benefit (Ostrowski and Pucko, 2022; Cesca et al., 2024). Further increasing the ROS levels beyond the level that is acceptable by glioblastoma cells, leads to cancer cell death, as indicated in our apoptosis flow cytometry and Western blot caspase-3 results (Cesca et al., 2024). Indeed, all currently used cancer therapies, including radiotherapy and chemotherapy, exert their anticancer effects through generation of ROS that are derived from the ionization or excitation of the water constituent of the cells, resulting in the formation of aqueous free radicals and ROS, including superoxide radicals and H_2_O_2_ (Olivier et al., 2021). However, the neurocognitive side effects of those currently used therapies on normal cells are pronounced (Lawrie et al., 2019; Jiang et al., 2023). Indeed, the exposure of healthy brain tissue to radiation or chemotherapy adversely affects brain plasticity and repair processes causing neurocognitive impairment (Lawrie et al., 2019). Therefore, the aspect of potential neurotoxicity should be considered when thinking of new therapies for glioblastoma.

Our study provides evidence that the pro-oxidative effect produced by CBD, celecoxib and 2,5-DMC or their combination exerted on glioblastoma cells, is accompanied by the minimal effects on normal astrocytes. Within the synergistic, and potentially available at the tumor site, concentrations (5 and 10 µM), the single compounds did not create significant cytotoxicity in HA cell line. When used in combination and at a concentration of 10 μM, they, however, reduced the percentage of living cells to ∼90%, which can still be regarded as relatively safe. In a recent study, where the neuron-astrocyte sandwich coculture were used to investigate the neurotoxicity of CBD, it was found that 15 and 30 µM CBD caused viability decrease and morphological damage in the neuron-alone culture, whereas these toxic effects were significantly attenuated by the support of astrocytes in the neuron-astrocyte coculture (Kim et al., 2023). However, in a study by Jurič et al., where the impact of submicromolar (0.1, 0.5, 1, and 5 µM) concentrations of CBD on perinatal rat cortical neurons and astrocytes were evaluated, it was found that during this critical period of time, CBD might induce toxic effects (Jurič et al., 2022). In this regard, the authors reported that in astrocytes, 0.5 µM CBD caused an immediate short-term dysregulation of ΔΨm, followed by ATP depletion with transient activation of caspase-8 and progressive activation of caspase-9 and caspase-3/7, leading to early apoptosis and subsequent necroptosis (Jurič et al., 2022).

The central signaling pathway linking oxidative stress and neuroinflammation is the nuclear factor-2 erythroid related factor-2 (Nrf2) pathway. Nrf2 is a transcription factor that regulates cellular redox status through endogenous antioxidant systems with simultaneous anti-inflammatory activity (Staurengo-Ferrari et al., 2019). Our study provides evidence, that glioblastoma cells when treated with CBD, celecoxib and 2,5-DMC activate the Nrf2 pathway-dependent antioxidant response, however the response is only around 25% stronger as compared to control cells. In this regard, the most pronounced effects were observed as a response to combinatory treatment, with both CBD + celecoxib and CBD + 2,5-DMC, especially when 10 µM concentration was used. In this case we observed upregulation of mRNA level of Nrf2, nuclear translocation of Nrf2 and increased binding to the Nrf2 consensus sequence. Similar results were also observed as a response to 10 µM CBD treatment, however, the in this case we did not find lower levels of cytosolic Nrf2 protein level, which usually accompanies higher levels of Nrf2 in the nucleus. Nevertheless, the ELISA test confirmed higher binding affinity of Nrf2 to DNA in cells treated with CBD, as compared to DMSO-treated cells. Moreover, the nuclear translocation was also detected following the treatment with 10 µM celecoxib and 10 µM 2,5-DMC, however without significant increase in the binding affinity of Nrf2.

Glial tumor cells exhibit high levels of ROS, but also high level of antioxidant response signaling pathways, including Nrf2 pathway. Constitutive Nrf2 activation is a hallmark of cancer cells and promotes their migration through accumulation of the BACH1 transcriptional regulator (Occhiuto et al., 2023). Whether or not it is better to activate or to inhibit Nrf2 pathway during glioblastoma treatment remains elusive. Vast majority of studies provides evidence that inhibiting Nrf2 pathway is a better option, as it can sensitize glioblastoma cells to chemotherapy and radiation treatments (Rocha et al., 2020; Awuah et al., 2022). However, recent advances in the understanding of Nrf2 go beyond the classical understanding. Currently, studies reveal the potential clinical application of Nrf2 activators in glioblastoma treatment (Kim and Jeon, 2022). In this regard, Nrf2 activators were shown to inhibit proliferation and induce differentiation and apoptosis in various cancer cells, including glioblastoma (Tsai et al., 2021; Kim and Jeon, 2022). In this context, RTA 404, a new trifluoroethylamide derivative of 2-cyano-3,12-dioxoolean-1,9-dien-28-oic acid was found to inhibit proliferation, cell migration, cell cycle progression, and induce apoptosis in GBM840 and U87 MG cells *in vitro*, possibly through its inhibition of N-cadherin and E-cadherin expression via its inhibition of the AKT pathway (Tsai et al., 2021). Nrf2 activators, such as dimethyl fumarate, are tested in clinical trials, in the context of glioblastoma (Talebi et al., 2023). Moreover, recently, de Sousa et al. found that high levels of Nrf2 sensitize temozolomide-resistant glioblastoma cells to ferroptosis via ABCC1/MRP1 upregulation (de Souza et al., 2022). The complex nature of the Nrf2 pathway still awaits elucidation (Rybarczyk et al., 2023a).

Here we provide evidence, that oxidative stress generated by CBD + celecoxib, and CBD + 2,5-DMC, is accompanied by mild Nrf2 pathway activation. Similar results regarding celecoxib, however, in the context of cardiovascular disease, were published by Al-Rashed. In this study, the treatment of human endothelial cells with celecoxib led to COX-2 independent signaling via phosphorylation of AMPK, resulting in the nuclear translocation of Nrf2 and AMPK-CREB-Nrf2-dependent signaling activation (Al-Rashed et al., 2018). As far as CBD is concerned, our results align with a study of Singer et al. who found that CBD induces Nrf2 activation, which in turn induces antioxidant response genes expression (Singer et al., 2015). According to our best knowledge, the impact of 2,5-DMC on Nrf2 signaling in glioblastoma cells has not been evaluated by other research groups before.

Furthermore, we also show that in response to Nrf2 pathway activation following CBD, celecoxib and 2,5-DMC treatment, *SOD1* is being upregulated, potentially accelerating the conversion of the superoxide anion to H_2_O_2_ and oxygen (Wang et al., 2018). However, we also show, that *CAT*, encoding H_2_O_2_ neutralizing enzyme, is not being upregulated, which might explain the cytotoxicity of these compounds, despite the Nrf2 mild activation.

The results of our study also reveal an increase in the protein level of p62, in response to CBD, celecoxib and dual therapy of glioblastoma cells with these two agents. Additionally, we observed similar upregulation of p62 following the treatment with CBD + 2,5-DMC. p62 is the key protein of the non-canonical Nrf2 pathway, coordinating the ubiquitin-proteasome system and autophagy (Staurengo-Ferrari et al., 2019; Kumar et al., 2022). Overall, p62’s domains provide a scaffold that directs substrates to autophagosomes and facilitates the autophagic process (Kumar et al., 2022). Therefore, when autophagy is induced, a corresponding decrease in p62 levels is observed. Conversely, p62 accumulation indicate autophagy inhibition (Puissant et al., 2012). Taking into consideration that autophagy can be regarded as one of glioblastoma therapy resistance mechanisms, the observed increase in the protein level of p62 as a response to the treatment with CBD, celecoxib and 2,5-DMC is a promising result (Khan et al., 2021).

Recent studies indicate that CBD influences both autophagy and apoptosis, which aligns with our finding (Fu et al., 2023). Our results are, however, in contradiction with the data published by Giannotti et al., who found that low-dose CBD makes U-87 MG cells more vulnerable to cytotoxic effects, reducing cell viability and mitochondrial dynamics while increasing autophagic flux and redox systems (Giannotti et al., 2025). The discrepancy between our and Giannotti’s results might arise from different glioblastoma cell line used as a model. In our study, instead of autophagy, we observed apoptosis induction, as presented by caspase-3 Western blot results. In this context, all analyzed compounds and combinations significantly upregulated caspase-3, indicating an ongoing apoptotic degradation of cells.

In addition to p62, we also analyzed LC3 protein, a widely recognized marker of autophagy. We observed accumulation of LC3 upon treatment with CBD, celecoxib, 2,5-DMC and its combinations, suggesting that autophagic processes are being triggered in U-138 MG cells. Interestingly, the increase in LC3 was accompanied by elevated p62 levels, which may indicate a blockade of autophagic flux rather than its full activation. Such a scenario has been reported in glioblastoma cells previously and is thought to contribute to apoptosis induction rather than to survival (Puissant et al., 2012; Khan et al., 2021). This dual regulation of LC3 and p62 points to a complex balance between autophagy and apoptosis following treatment with the investigated compounds.

We also evaluated the component of Akt/mTOR pathway, a critical negative regulator of autophagy. Treatment with CBD and its combinations reduced levels of mTOR protein expression, consistent with the accumulation of LC3 and suggesting suppression of the Akt/mTOR axis. Given that mTOR inhibition promotes autophagy initiation, our findings indicate that the compounds may trigger autophagic signaling, but incomplete flux - as evidenced by p62 accumulation - may redirect cells toward apoptosis instead of survival. This interplay between mTOR suppression, p62 accumulation, and LC3 induction highlights a possible mechanism by which CBD, celecoxib, and 2,5-DMC tilt the balance from cytoprotective autophagy toward apoptotic cell death in glioblastoma.

Eventually we would like to point that the combination treatment with CBD + celecoxib, but also CBD + 2,5-DMC influence cancer cell migration. In this regard, all 10 µM solutions of CBD, celecoxib and 2,5-DMC and their combinations significantly halted cell migratory potential. This might result from the anti-inflammatory properties of celecoxib, or the Nrf2 pathway modulation by all three compounds, since this pathway is also involved in migration and invasion of glioma cells (Pan et al., 2013).

In conclusion, the combination of CBD with celecoxib or 2,5-DMC represents a promising therapeutic strategy for glioblastoma. While CBD alone induces cytotoxicity, ROS production, and apoptosis, our synergy analysis demonstrates that combining CBD with celecoxib or 2,5-DMC allows effective killing of GBM cells at lower CBD concentrations. This dose reduction could potentially reduce neurotoxicity risks. Thus, by leveraging the synergistic effects of these compounds, it may be possible to overcome the challenges of therapy resistance, while providing lowest-possible side effects for glioblastoma patients. Future studies should focus on validating these findings in animal models and exploring their clinical applicability.

## Data Availability

The original contributions presented in the study are included in the article/supplementary material, further inquiries can be directed to the corresponding author.

## Footnotes

1
https://www.synergyfinderplus.org/
